# Assessing young unmarried men's access to reproductive health information and services in rural India

**DOI:** 10.1186/1471-2458-11-476

**Published:** 2011-06-17

**Authors:** Arundhati Char, Minna Saavala, Teija Kulmala

**Affiliations:** 1 School of Health Sciences, University of Tampere, 33014 Finland; 2 Vaestoliitto, The Family Federation of Finland, Helsinki, Finland; 3 The Department of International Health, University of Tampere, 33014, Finland; 4 Department of Social Research, University of Helsinki, Finland

**Keywords:** Young men, sexual and reproductive health, access, services, information, condoms, rural India

## Abstract

**Background:**

We investigated the accessibility of reproductive health information and contraceptives in a relatively less developed area of rural central India and assessed the risks facing young unmarried men.

**Methods:**

This cross-sectional study used both qualitative and quantitative methods. Participants included 38 unmarried rural men in four focus-group discussions and a representative sample of 316 similarly profiled men, aged 17-22 years, in a survey. Information was collected on the men's socioeconomic characteristics; awareness, knowledge, and perceptions of family planning; attitudes toward future contraceptive use; intra-family communication; knowledge about STIs/HIV/AIDS; and access and use of condoms. Content analysis for qualitative information and descriptive analysis for survey data were used to draw conclusions.

**Results:**

Young unmarried rural Indian men's sexual and reproductive health (SRH) knowledge is limited, although the majority is familiar with condoms (99%). The young men identified electronic mass media (67%) as the prime source of reproductive health information, yet they lacked detailed knowledge of various contraceptives and felt ignored by health providers, who, they felt, would be capable of providing SRH information through interpersonal communication. Young men are more concerned about avoiding infections and securing sexual pleasure and less concerned about avoiding potential pregnancies. For example, 68% of the young men were aware of condoms and their HIV/AIDS preventive role, but only about two-fifths mentioned condom use to prevent unwanted pregnancies. Although most young men (96%) knew where to access a condom, they felt uncomfortable or embarrassed doing so in their own villages or close by because of socio-cultural norms that prevented them from using contraceptives. Very few respondents (4%) disclosed using condoms themselves, but 59% said they knew someone from their peer group who had used them.

**Conclusions:**

Young unmarried men in rural India are underserved with regard to SRH information and services, because they are not recognized as key targets under the public health system, and they receive their limited knowledge and information mainly from the mass media; this situation could be greatly improved by public health service providers. It is important that programmers involve young men with effective communication strategies to enable them to act responsibly with regard to their own sexual health needs.

## Background

Adolescents and young adults form one of the largest groups with an unmet need for reproductive health services in South Asia [1,2]. India has committed itself to a comprehensive family-planning program, which includes providing reproductive health information to all population groups, regardless of gender or age [3]. India has the world's biggest-ever youth generation, with an estimated 300 million young people (aged 10-24 years), comprising almost one-third of the country's population [4]. The enhanced programmatic focus on young people's reproductive health services and information needs requires more detailed culture-specific information on reproductive health and reproductive choices [5].

The sexual and reproductive health (SRH) needs of young unmarried people differ from those of young married people in significant ways that have thus far been poorly understood and addressed in India. In the socio-cultural context of South Asia, premarital sex is censured not only for girls, but also for boys, and consequently young people's sexual activity and sexual health information and needs remain largely unaddressed. Not only urban but also rural Indian adolescents spend an increasingly longer time at school, experience puberty at a younger age, and marry and have children later than in the past. Neglecting this group will have major future implications, since the sexual behavior during adolescence will have far-reaching effects as these young people progress into adulthood. In South Asia, over the last decade, both researchers and governments have begun to shed traditional inhibitions toward young people's SRH, and a growing body of empirical evidence combined with government interest has provided opportunities to take stock of the regional situation in this regard. Studies have consistently found that young people in developing countries lack basic knowledge about sexuality and contraception [6-11]. Moreover, even subjects in Indian studies who report awareness tend to harbor misperceptions or possess only superficial information about these issues [1,12].

Most available studies about unmarried youths have focused on sexual behavior in the context of HIV/AIDS [13-17]. The few studies that do address young people's reproductive health and sexuality are mostly studies of women, with very limited emphasis on young unmarried men [18-20]. Those focusing on young males tend to concentrate on urban populations, particularly college students [21-24]. The present study targeted young unmarried men aged 17-22 years in a rural context, a group thus far rarely addressed in research, despite the fact that two-thirds of the Indian population still lives in rural areas. Young men in this age-group constitute about 16% of India's population, and are on the threshold of a life-cycle milestone-marriage. In rural India, 68% of men aged 15-45 years are ever-married. Only 4% of rural men marry very early, by age 19 and one-third are married by the median age of 21.5. Only 1% of males in the age-group 45-49 have never been married, indicating that marriage is still nearly universal in India [12]. In addition to affecting young men's and their current partners' opportunities to enjoy sex responsibly and safely, young unmarried men's current views on SRH will affect them and their spouses in the future.

### Justification for the study

During the years 2001 to 2005, DKT India, a contraceptive social marketing organization, carried out an extensive reproductive health intervention in rural areas of the state of Madhya Pradesh in central India. The principle objective of this intervention was to inform and educate the rural communities, which included men and women in the reproductive age-groups of 15-45 years on various reproductive health issues, including family planning. The intervention used various communication media, including interpersonal communication, street theater, and audiovisual as well as print media, for effective impact of reproductive health messages.

During the course of this intervention, young unmarried men aged 17 to 22 freely discussed various reproductive health issues. However, the subjects lacked adequate knowledge of contraceptives, although they had heard about them, and they indicated they were not very comfortable about accessing contraceptives.

It was therefore important to investigate this sector of the population in terms of individuals' access to contraceptives and the problems they face with regard to information and access to reproductive health commodities. This study investigates whether young unmarried rural men in India are underserved in terms of SRH issues. We hypothesize that their knowledge and access to SRH information and services remain inadequate, due to limited interaction with health-care providers, with the mass media being their main channel of information. We review their knowledge, attitudes, and perceptions about SRH. The findings from this study will address key areas of youth SRH in terms of access to information and services, and will help direct effective communication strategies for this target audience.

## Methods

The study was conducted among unmarried young men aged 17-22 years from two rural districts in Madhya Pradesh.

### The study setting

Madhya Pradesh has a total population of 60.4 million, of which about 73% reside in rural areas. The total fertility rate is 3.12 for the state overall and 3.34 for rural areas; these figures are higher than the comparable national rates of 2.68 and 2.98, respectively [12]. About 70% of Madhya Pradesh residents depend on agriculture for their income. The sex ratio at birth is 933 females per 1,000 males; the birth rate is 32.3 per 1,000 persons, and about 50% of women and 70% of men are literate [4]. The average age at marriage is 21.7 years for males and 18.4 years for females. About 64% of rural men aged 25-29 years were married by age 21 and 66.5% women aged 20-24 years were married by age 18 [25].

The two study districts, Sehore and Raisen, have a total population of around 2,200,000, of which about 10% are males in the age-group 17-22. Of these, approximately half are unmarried, which would make the size of the target group approximately 110,000 [4].

### Data

The data was drawn from a larger study, "Male Involvement in Sexual and Reproductive Health in Central India," conducted to examine how rural Indian men act to secure their own and their partners' SRH. The results of the larger study were intended to have relevance for SRH policies: to identify strategies for positively influencing men's SRH-seeking behavior, including participation in fertility regulation, practice of safe sex, support of maternal and child health, and prevention of unwanted pregnancies. We used a multi-method approach, including focus-group discussions and a cross-sectional survey, because we believed that the study of such an intimate and partly taboo subject would most likely benefit by adopting different methods. The qualitative data was used heuristically to improve the survey instrument and, later, to assess and interpret the survey results. In addition, the survey enabled the findings that emerged from the focus groups to be corroborated and assessed. Combining the two methods also helped to bring into focus the varied social aspects of young people's fertility, and broadened our understanding of the social mechanisms of demographic phenomena, which are often difficult to capture through surveys alone [26].

Data collection occurred in two phases from March 2005 to September 2005: initially, there were focus-group discussions; these were followed by the cross-sectional survey. Currently unmarried 17-to 22-year-old men were eligible to participate in the study.

#### Focus-group discussions

We used snowball sampling as a recruitment technique to gather participants for the focus-group discussions (FGDs), a relatively homogeneous group of unmarried men aged 17 to 22 years, who would provide credible information about practices and norms with regard to youth SRH. Despite snowballing having its limitations as a recruitment technique, it was chosen because the topic of the discussions was sensitive, and this approach required making use of personal networks and encouraging the young men to overcome their apprehensions and attend the group discussions. Since the initial main purpose of the FGDs was to direct development of the survey questionnaire, it was restricted to one district, Sehore. Out of 66 villages in Sehore with a population of at least 1000, four were randomly selected based on their location in relation to the main road. Two of these were chosen from within a 10-km radius of the road, while two were outside this radius. Only one FGD per village was carried out, to ensure that the participants were not affected by rumors about the discussion spreading in the village and leading to information bias. Young men in the age-group of 17-22 years were recruited from each selected village: one such individual was contacted and briefed about the purpose of the group discussion, and he initiated the recruitment process. Overall, 38 men were contacted, and all agreed to participate in the four FGDs after the details of the study were explained to each; their informed consent was obtained.

The four discussions reached a saturation point, and further discussions were not expected to bring up any new points of view. FGDs aim at deciphering commonly shared convictions and attitudes, not the variation in individual experiences, and we believe that carrying out the discussions in other villages or districts of the state would most likely have produced a similar range of views, possibly textured with local idiosyncrasies. As is generally the case, the qualitative data was not intended to be representative. It is possible that there was a selection bias among those attending the groups; however, we consider this unlikely considering the background characteristics of the participants. The most important factor ensuring reliability of the qualitative data was that the discussions were conducted in an atmosphere of trust and sharing. The principal investigator, who has long field experience working in the district and fluency in the local dialect, could not detect any factors that might potentially have rendered the discussions systematically biased or unreliable.

The discussion guide used for the FGD was piloted in a village not included in the final village selection, and the guide was finalized before being employed in the study. Two highly experienced male research associates moderated the discussions. As the principal moderator directed the discussions, the co-moderator noted nonverbal cues. Discussions were conducted in the local language, Hindi, and lasted approximately two hours. All the FGDs were held in the community center of the village, which ensured that there was no disturbance or outside noise while they were being conducted. All the discussions were recorded, transcribed verbatim in Hindi, and then translated into English. To validate the tapes, we checked them against the translated text for any inaccuracies. Data was analyzed by qualitative content analysis, using ATLAS.ti software for Windows version 5.0. (Scientific Software Development GmbH). Topics discussed during the FGDs were young unmarried men's knowledge, attitudes, and perceptions about SRH, including family planning, access to reproductive health services and information about contraceptives, and sexually transmitted infections (STIs)/HIV/AIDS.

#### Profile of FGD participants

The average respondent age across the four village groups was around 18 years, and most men had had at least eight years of schooling. Three men were Muslims, and the rest were Hindus. Among the Hindus, the majority (21) belonged to the caste administratively designated as Other Backward Class; nine belonged to the Upper Class, and the remaining five were of the Scheduled Class. All participants were residents of the village in which the discussions were conducted.

#### Cross-sectional survey

The second, main part of data collection involved a cross-sectional survey in Sehore and Raisen. A three-stage probability sampling procedure was used to select villages, households, and eligible persons as sampling units at each stage of the survey. From each district, villages with a population of 1000 to 3000 (as per the census listing) were drawn up; the decision to select larger villages was based mainly on logistical reasons. There were 137 such villages. For each of these villages, we obtained the best available estimates of female literacy [4]. The villages in the two districts were divided into low, medium, and high literacy levels; two villages were randomly selected from each group, resulting in a total of 12 villages, six from each district, using a probability-proportional-to-size (PPS) sampling method. This process ensured heterogeneity in the data, which is essential to obtain meaningful results. If a particular selected village was one where the FGDs had been held with similarly profiled men, the next village on the list was selected; this ensured that the survey was not conducted in the same villages as those that held the FGDs and avoided any information bias. Next, a house listing was drawn up to identify households that had unmarried men in the 17-22 age-group. Survey household selection was carried out using a systematic random-sampling method, and every third household was chosen. In any given household, only one eligible respondent was randomly recruited into the study.

Six male postgraduate students with backgrounds in the social sciences were recruited from the local state university as research investigators. The selected researchers were rigorously trained by the principal investigator for four days, including one day of field training. Of these investigators, two were identified as supervisors for the fieldwork, and they were specially trained in sampling methods and selection of respondents for the survey. Once trained, the research investigators conducted a household survey.

A semi structured interview schedule was used to collect information from the young unmarried men. The survey instrument was pretested extensively in areas similar to but not contiguous with the study sites, and it was later finalized by modifying, adding, or deleting questions. Information was collected on the subjects' socioeconomic characteristics; knowledge, awareness and perceptions of family planning; attitudes toward contraceptive use; intra-family communication and knowledge about STIs and HIV/AIDS; and access to and use of condoms. Survey questionnaire topics were decided based on issues emerging from the FGDs that preceded the survey. About 20% of the questions were open ended and later coded for analysis. Data collection was conducted during September 2005 for one month; 316 men were interviewed.

Since the interviews required privacy, the investigators took the respondents outside their homes; if necessary, the respondents were taken to another village location, such as under a tree off the road or into the fields close to the village, where they would not be disturbed during the questionnaire administration. Each participant gave oral informed consent after being apprised of the purpose of the research. A 10% back-check of questionnaires in each village was carried out by the research supervisors, who also scrutinized all questionnaires on the same day that they were filled to check for completeness, consistencies, and clarity of marked codes. Data were managed and analysis was performed using the SPSS statistical package for Windows, version 13.0 (SPSS Inc., Chicago IL).

The study was approved by the Institutional Review Board of Tampere School of Health Sciences and the Ethics Board of the International Institute for Population Sciences in India. The study was conducted in accordance with the Helsinki Declaration of 1975, as revised in 2000.

## Results

### Background characteristics

The young men surveyed were on average 19-years-old. The majority (90%) were educated to at least middle-school level (eight years schooling). Hindus were predominant among the interviewed young men. Most of them belonged to the economically and socially weaker classes, with 60% coming from castes administratively designated as Other Backward Classes. About three-fifths of the young men were employed in the agricultural sector, either as cultivators of their own land or as laborers. Less than two-fifths of the young men were engaged in nonagricultural activities. Only 3% of the young men were not employed in any income-generating activity at the time of the survey. About half of the respondents reported living in a nuclear family, with parents and unmarried siblings (Table 1).

**Table 1:** Background characteristics of young unmarried men aged 17 to 22 years, by selected sociodemographic characteristics, Madhya Pradesh, India, 2005 (n = 316)

Characteristics	Categories	N (%)
**Age (years)**		
*Mean age(+ SD)*	19.2 (± 1.68)	
	17-19	187 (59.1)
	20-22	129 (40.9)
**Education**	None	16 (5.1)
	Primary	13 (4.1)
	Middle	65 (20.6)
	Secondary+	222 (70.2)
**Religion**	Hindu	295 (93.4)
	Muslim	20 (6.3)
	Other	1 (0.3)
**Caste**	Upper Caste	72 (22.8)
	Scheduled Caste	37 (11.7)
	Scheduled Tribe	20 (6.3)
	Other Backward Caste	186 (58.8)
**Occupation**	Agricultural based	186 (58.8)
	Non-agricultural based#	120 (38.0)
	Unemployed	10 (3.2)
**Monthly family Income**	Low (up to Rs.1,000)	53 (16.8)
	Medium (Rs.1,000-3,000)	182 (57.6)
	High (> Rs.2,500)	81 (25.6)
**Type of family**	Nuclear	163 (51.6)
	Joint	153 (48.4)
**Average family size**		6.5

### Family-planning knowledge

The survey showed that young unmarried men had limited knowledge of SRH issues. However, almost all the subjects knew about condoms. Knowledge about family planning (*parivar niyojan*) was elicited from the males in the survey ("Have you heard of the method/technique with which a couple can either delay or avoid becoming pregnant?"). For those who answered yes, the next question was an open-ended one: "What does family planning mean to you?" In the Indian context, "family planning" or its local language equivalent refers to contraceptive methods; among married men and women, it particularly refers to female sterilization, which is the most commonly used method [27,28].

Table 2 shows the percentage of respondents who had heard of a method for delaying, spacing out, or preventing pregnancies, as well as those who could spontaneously name individual methods of contraception. Overall, 98% of the subjects had heard of some family-planning method. Condoms were the most widely known method, and nearly every young man (99%) mentioned them. Contraceptive pills were the next-most commonly named method (85%). About one in five young men mentioned the intrauterine device (IUD). As noted above, female sterilization is the most popularly known and used method among married couples in India [12]. This is true also among rural married couples [27]. However, knowledge of this commonly known and used method was low, and only about 8% of the young men mentioned female sterilization. Also, natural family-planning methods, such as abstinence and withdrawal, were hardly mentioned among this group of respondents. The average number of methods that respondents mentioned was two.

**Table 2:** Knowledge of family planning among 316 young men aged 17-22 years in rural central India, 2005

Description	N (%)
Had heard of methods for delaying or stopping pregnancies (Yes)	308 (97.5)

Family planning methods heard of by respondent*	
Condoms	305 (99.0)
Oral contraceptive pills	261 (84.7)
Intra Uterine Device (IUD)	59 (19.2)
Injectable contraceptive	30 (9.7)
Female sterilization	23 (7.5)
Male sterilization	15 (4.9)
Abstinence	2 (< 1.0)
Withdrawal method	1 (< 1.0)

Average number of modern methods cited	2.0

* Percentage may exceed 100.0 due to multiple responses.

### Attitude toward condoms and access to them

As mentioned above, the most often mentioned contraceptive method among the young men was condoms. Young men considered condom use more in terms of pleasure and disease prevention and only secondarily in terms of pregnancy prevention. While almost 70% were aware of the use of condoms as protection against HIV/AIDS, only about two-fifths reported that condoms were used to prevent pregnancies. Further, most men (96%) knew where to obtain condoms. Most commonly, they mentioned health centers as a source of condoms, and more than one in two mentioned shops (both general stores and pharmacies) (Table 3).

**Table 3:** Young unmarried men's knowledge about and attitude toward condoms in rural central India, 2005

Description	N (%)
Has heard of condoms (Yes)	305 (99.0)

Why condoms are used *	
To prevent HIV/AIDS	207 (67.8)
To prevent pregnancies	115 (38.7)
To space out pregnancies	26 (8.5)

Knows where to get/buy a condom (access)*	293 (96.0)

**Sources of condom access**	
Health center	212 (72.3)
Shop/pharmacy	152 (51.9)
Government hospital	61 (20.8)

* Percentage may exceed 100.0 due to multiple responses.

Although the subjects were aware of where condoms could be obtained, they felt uncomfortable in accessing them in their villages or close to home because of social and cultural norms that prevented them from using contraceptives. The dialogue below details how the cultural context makes contraceptives inaccessible to this group, even though they are easily available in the community.

Respondent (R)2: The nurse-midwife only goes to see married people and women who are pregnant. Who comes to ask what we want? We're very shy to even approach these people for a condom. It would mean that we were doing something wrong.

Moderator (M): Do you know if they are available in the market?

R5: Yes, they're available in the stores in our village, as well as at the shops in towns and cities.

M: So would you go to the store to purchase one?

R5: Yes, but only in the big city. Not here (in our village shop). If someone saw me buying a condom, word would spread. (FGD 4)

Clearly, young men seemed to be thinking of these issues, and they had ready opinions on the use of condoms. The overarching moral condemnation and its effects on discussing the issues also came through forcefully.

### Condom use

Although the survey was intended to try to gather information about whether the respondents themselves had used condoms, very few (12 men; 4%) reported having done so. However, a large number reported knowing a friend or peer that had used a condom. It was our impression that at least some of the respondents may have reported their own experience as that of another person.

Though the subjects did not readily admit experience with condom use, such issues were much more openly discussed in the focus groups than in the survey:

M: Have you used one?

R1: Never needed to. We can do our "work" [sexual intercourse] without one.

M: Without a condom, what do you do?

R1: With some girl... *(laughs)*

R2: Some people do. We don't. Some people must be doing it in the village.

R3: My brother did it. He's sitting there.

M: Did you use a condom? (Directing the question to the person indicated by R3)

R4: I did use a condom once. It tore *(laughs)*. I established sexual relations. I thought that I would get AIDS or something, so I'd better use a condom. But then it tore.

M: What happened then?

R4: Nothing happened. It tore, and I went home. (FGD 1)

As seen in the above excerpt and also in the survey data regarding knowledge about condoms, condom use among the young unmarried men was mainly understood in the context of pleasure and was motivated by the desire to avoid infections; the contraceptive function was clearly secondary.

Analysis revealed that about three-fifths of respondents in the survey knew of someone who had used a condom. About two-fifths of the subjects reported being told of a positive experience with a condom. Although during the focus groups, the young men discussed their experience with condom use, in the survey they reported very little discussion taking place in the community with regard to condoms (46%). Only 17% of the young men mentioned any open or free discussion in the community about condoms. Again, friends were the people they trusted most and the ones they thought they would feel comfortable about discussing condoms with in the future (for 90% of the young men). Only 2% of the young men mentioned planning to discuss condoms with a future wife (Table 4).

**Table 4:** Young unmarried men's views in community discussions of condom use in rural central India, 2005.

Description	N (%)
**How openly does the community discuss condoms?**	
There is very little discussion	146 (46.2)
Like-minded people/very close friends discuss	65 (20.6)
People discuss freely	54 (17.1)
Can't say	51 (16.1)
Total	316 (100.0)

**Who would you discuss condom use with?**	
Friends	286 (90.5)
Doctor/nurse	14 (4.4)
Future wife	9 (2.9)
No-one	7 (2.2)

Total	316 (100.0)

### Sources of reproductive health information

The main source of SRH information was the electronic mass media (67%; mostly television, but to a lesser extent radio). However, among the young men, there was a notable preference for personal, face-to-face information through health professionals (72%). About one-fourth of the young men mentioned friends and other village peers as people from whom they would feel comfortable obtaining information in the future. Family members were not mentioned as preferred sources in this regard, while only 2% of respondents preferred television as a future source of reproductive health information (Table 5).

**Table 5:** Sources of information about reproductive health among young men aged 17-22 years in rural central India, 2005

Reproductive health (RH) information	N (%)
Received information	308 (97.5)

**Principal source of RH information**	

Media (TV/Radio)	207 (67.2)
Health workers	73 (23.7)
Friends/village peers	26 (8.5)
Family	2 (0.65)
Total	308

**Preferred source of RH information in the future**	
Health workers	225 (72.1)
Friends/village peers	77 (24.7)
TV	6 (1.9)
Not sure	8 (2.5)

Total	316

During group discussions, the young men mentioned that the mass media was important in terms of maximum reach of family-planning messages. However, here too they stressed the importance of interpersonal communication for information on reproductive health, particularly contraception, through community health workers. The young men wished for more detailed information from health workers and a chance to discuss their queries in privacy:

"Auxiliary nurse-midwives come to talk to unmarried girls in our villages. The nurse-midwives sit in the *anganwadi *[a center catering to services for 0-6 year-olds] behind closed doors and talk to them for hours on end. Why is it that they don't come and talk to us?" (FGD 2)

"We too wish to be told about various contraceptives and how they should be used, and we want to clear up all our doubts about sexual issues. But where do we go for such information?" (FGD 2)

The young men also discussed the importance of schools and educational institutions as sources of information:

"Nowadays, most children from our villages go to schools to study. If such information could be given to older children in school, it would be well accepted and received, since teachers in our villages are highly respected." (FGD 3)

"Many youths from our villages go to town for further studies. Teachers can give information about various family-planning methods available, their use, and information about access. This will be useful to everyone." (FGD 1)

The qualitative data shows that young unmarried men in rural India regard schools and educational institutions as acceptable, even desirable, channels of SRH information.

### Communication about family planning

Clearly, the role of the peer group is important for the young men. They would also prefer to receive information from service providers, although this is not easily available. In an attempt to understand whether, after accessing information, young people ever discussed family planning-and if so, with whom-various questions were used in the survey (Table 6). Slightly more than one in two young men responded that he had indeed discussed family planning with someone. Friends or peers seemed to be the most popular choice of people with whom they discussed family-planning issues. On being queried about the people with whom they would prefer to discuss reproductive health matters in the future, again friends seemed to be most popular choice. Only 14 men (4%) mentioned that they would discuss such issues with their future wives.

**Table 6:** Young men and communication about family planning in rural central India, 2005

Description	N (%)
Discussed FP with someone (Yes)	169 (53.5)

**Discussion partners**	
Friends	130 (76.9)
Health workers	20 (11.8)
Family members	14 (8.3)
Villagers	5 (3.0)
Total	169

**Preferred discussion partner for discussing FP in future**	
Friends/other village peers	182 (57.6)
Doctor	82 (25.9)
Health workers	24 (7.6)
Wife (after marriage)	14 (4.4)
Family (older brother or mother)	8 (2.5)
Not sure	6 (1.9)

Total	316

The young men in the focus groups were also queried about whom they preferred for discussing SRH issues. Above anyone else, they preferred to discuss such issues with their friends, as clearly emerged from the group discussions:

M: Who do you prefer to discuss your sexual health with?

R1: I discuss that kind of thing very openly with my friends.

M: So what do you talk about?

R3: It may start like this: someone tells a friend that he has some kind of problem. That friend tells me. I tell another friend, and he tells someone else. It spreads like that. Everyone gets to know. So we're aware that such problems can occur to any one of us, and we tell the first friend how to get treatment.

R2: Like for example, he (*pointing to one of the participants*) told me that he has some kind of boils on his private parts. So I told his older brother about the problem and asked him to get his brother treated. So his family got him treated. (FGD 3)

## Discussion

Our findings point out the need to develop effective strategies to educate young unmarried men in rural India about reproductive health and contraception, and also to improve their access to family-planning information and services, thus reducing the risk of unwanted pregnancies and STIs. The age of marriage among rural men is rising; thus the period after puberty when they are most susceptible to unwanted pregnancies and STIs is increasing. For example, the pressure to achieve financial stability before marriage often forces young men to delay marriage, which increases the risk of unsafe sexual activity if they are not provided with accurate information and appropriate services [6]. At the same time, young men at this stage are curious and willing to absorb new information. A lack of the necessary reproductive health information or services during this period will be detrimental to the reproductive health of young men and married couples.

Overall, the results indicated a good level of knowledge about the existence and effects of condoms. However, little information was available to the young men on other methods, such as IUDs, injectable contraceptives, or even the most common family-planning method in rural India, female sterilization. With the Indian government's promotion of the use of condoms as a result of HIV/AIDS [29,30], it is not surprising that most young men have heard about this form of contraception and are more comfortable talking about it than about any other method. Since the only available contraceptives for young men in rural India are condoms, it is natural that in the group discussions the subject was not so much one of contraceptives generally than of condoms alone. Also, it was clear that among this group, condoms are discussed more in the context of pleasure and as protection against infections for both parties than to avoid pregnancies. It is important that the responsibilities of these young men, both in terms of health and enjoyment for themselves and their sexual partners, be stressed to them. Communication campaigns should emphasize that the dual merits of condoms are prevention of both STIs/HIV and pregnancies.

Young people face many barriers in accessing services in preventing HIV and unwanted pregnancies. Although national SRH and HIV programs recognize the need to provide such services, concerted action is often hindered by a lack of clear understanding on how to reach young people with the information and services they need. There is often also a level of discomfort about providing young people with such services [31]. A review of various published and unpublished studies has documented the increased use by young people of health services with trained providers, along with increased availability and accessibility of youth-friendly services [32]. Within the Indian family-welfare system, health workers target only married couples with SRH information and services. The FGDs clearly showed that young unmarried men are not targeted with any such information, nor is there any available assistance as to where they might seek services relating to SRH. The little information young men possess comes from television and other mass media. Though television is an important source of family-planning information because of its wide reach, it does not help resolve individual SRH queries, since such media offer generalized information, and this may be insufficient for young men. They wish for more interpersonal communication with credible sources, such as community health workers. The discrepancy between the principal source of information (electronic mass media) and its low status as a preferred source of information among young unmarried men is noteworthy. There is clearly a need for other sources of information on SRH besides that provided by television and other mass media.

Another important source of SRH information, as suggested by the present study, is schools and other educational institutions. With universal education becoming an important program for the Indian government under its campaign "Education for all," more and more young people enroll in schools and complete their school education. A mixture of mass media and school education programs could help improve the information provided to young people.

Only 4% of survey respondents reported contraceptive use, although there were more indications of experience, especially with condoms, during the FGDs. By and large, the young men did not seem very open about their own contraceptive experience. This may be a result of the moral condemnation of premarital sex for both males and females in Indian society. Both the FGD and survey data point to limited reproductive knowledge among young unmarried men. They seem to be unaware or indifferent to the effects of unprotected intercourse on their sexual partners in the form of unwanted pregnancy. For the majority of the young men, contraception means condoms, and their reason for using them is to avoid contracting HIV. This view may partly reflect the fact that if young unmarried men do have sex, it is most probably with sex workers or older married women, and only secondarily with adolescent girls [33]. The almost universal awareness of condoms and HIV/AIDS among young unmarried rural men is a good beginning, but it is a grossly insufficient basis for responsible and safe, present and future sexual behavior. Though evidence of sexual risk taking is not available at the national level, a synthesis of small and admittedly unrepresentative studies undertaken in different geographic settings and among different subpopulations of young people suggests that 15-30% of young men and fewer than 10% of young women have engaged in premarital sexual relations, mostly unprotected [34]. This study confirms that premarital sex among young unmarried Indian men is not as rare as is commonly believed. Three out of five respondents revealed knowing other peers who had used condoms.

The only contraceptive that is suitable for young unmarried men-condoms-is also inaccessible to them in their immediate geographic area. Clearly, young men lack basic access to condoms more because of socio-cultural considerations than practical availability. The men argued that while it is easy for married people to have access to condoms, young unmarried men find it difficult to do so in the vicinity of their villages, and they would rather buy them from shops outside the villages-away from the prying eyes of their village peers/elders. This was also observed in the discussion by Meirik [35].

Unsurprisingly, very few young men mentioned any plans to discuss family planning with a future wife. They preferred discussing the matter with friends. This brings up yet another important issue: future communication between spouses among these soon-to-be-married young men. This group of young unmarried men does not seem to be aware of or believe in the importance of having an open discussion with their future spouses. However, their present intention not to discuss condoms with their wives does not necessarily determine their eventual actions once married. In the Indian context, marriage is a watershed moment in young men's lives and could change many of their views concerning women.

## Conclusions

The findings clearly indicate the need for focused interventions in the rural areas of India, where young men seem ready and willing to absorb reproductive health messages and access services. It is important that program planners identify this underserved group of young unmarried men with effective communication strategies that will enable them to act responsibly not only in the present, but also in the future, when they are married, and take crucial family-planning decisions together with their wives. Youth-inclusive communication campaigns, with more focused intervention targeting young unmarried men, should be developed, and health workers should be made aware of the needs of this group in future communication.

## Competing interests

The authors declare that they have no competing interests.

## Authors' contributions

All three authors-AC, MS, and TK-participated and contributed in the conception, design, analysis and writing of the article. Further, all three authors read and approved the final manuscript, and are aware that the manuscript is being submitted to the journal.

## Pre-publication history

The pre-publication history for this paper can be accessed here:

http://www.biomedcentral.com/1471-2458/11/476/prepub
